# Visuomotor adaptation in head-mounted virtual reality versus conventional training

**DOI:** 10.1038/srep45469

**Published:** 2017-04-04

**Authors:** J. M. Anglin, T. Sugiyama, S.-L. Liew

**Affiliations:** 1 Chan Division of Occupational Science and Occupational Therapy, Division of Biokinesiology and Physical Therapy, Department of Neurology, Stevens Neuroimaging and Informatics Institute, University of Southern California, Los Angeles, CA, USA

## Abstract

Immersive, head-mounted virtual reality (HMD-VR) provides a unique opportunity to understand how changes in sensory environments affect motor learning. However, potential differences in mechanisms of motor learning and adaptation in HMD-VR versus a conventional training (CT) environment have not been extensively explored. Here, we investigated whether adaptation on a visuomotor rotation task in HMD-VR yields similar adaptation effects in CT and whether these effects are achieved through similar mechanisms. Specifically, recent work has shown that visuomotor adaptation may occur via both an implicit, error-based internal model and a more cognitive, explicit strategic component. We sought to measure both overall adaptation and balance between implicit and explicit mechanisms in HMD-VR versus CT. Twenty-four healthy individuals were placed in either HMD-VR or CT and trained on an identical visuomotor adaptation task that measured both implicit and explicit components. Our results showed that the overall timecourse of adaption was similar in both HMD-VR and CT. However, HMD-VR participants utilized a greater cognitive strategy than CT, while CT participants engaged in greater implicit learning. These results suggest that while both conditions produce similar results in overall adaptation, the mechanisms by which visuomotor adaption occurs in HMD-VR appear to be more reliant on cognitive strategies.

Virtual reality (VR) provides a computer-generated environment that allows users to engage in virtual experiences. VR has been used for simulations and training across a wide range of disciplines including military, education, and healthcare applications. While previous versions of VR have traditionally consisted of a participant sitting in front of a large screen, exciting recent technological advances have produced new forms of VR, such as head-mounted displays (HMD), which allow for more immersive experiences. HMDs (e.g., Oculus Rift, HTC Vive) have recently become commercially available and affordable and are thought to induce greater feelings of embodiment and immersion compared to traditional forms of VR^1^. VR using head-mounted displays (HMD-VR) thus provides a novel medium for examining the effects of context and experience on human behavior.

Previous research has shown benefits of VR in healthcare, specifically for motor rehabilitation. Traditional VR, which uses large monitors and motion capture equipment, such as the Microsoft Kinect or Wii, has been used to improve clinical motor performance in individuals with stroke and cerebral palsy^2^,^3^, and shown positive effects, attributed to increased motivation and participation^2^,^4^,^5^. Evidence suggests that some forms of VR in rehabilitation can produce similar^4^,^6^,^7^ or greater^8^ therapeutic gains as compared to conventional rehabilitation, and the use of VR in motor rehabilitation has led to improved motor recovery and mobility-related outcomes^8^,^9^,^10^,^11^. However, certain patient characteristics, such as one’s severity of motor impairment or the amount of time since the loss of motor function, can affect how much a patient benefits from VR-based interventions^12^,^13^.

Aside from the use of VR to improve rehabilitation gains by increasing patient motivation and engagement in a task, VR, especially HMD-VR, could be a powerful tool to experimentally manipulate context and feedback, and examine these effects on human motor adaptation, learning, and recovery. HMD-VR can provide a similarly engaging and motivating environment with the additional potential to increase one’s feeling of embodiment in the virtual environment^14^,^15^, which may have additional effects on motor performance. For example, healthy individuals that are trained in HMD-VR with a body that has longer arms than their own learn to interact with the world as though they have longer arms, and this behavior persists briefly outside of the HMD-VR environment^16^. Similarly, healthy adults that are shown to have a child’s body in HMD-VR begin to exhibit more childlike behaviors than adults shown an adult body^17^. Thus, embodiment through HMD-VR may be a powerful tool for manipulating and enhancing motor learning by showing individuals movements in HMD-VR that they are not able to perform in reality.

However, before jumping straight into using HMD-VR for motor learning and rehabilitation, it is important to understand the potential differences and mechanisms underlying motor performance between these two environments so that virtual conditions can be tailored to maximize learning and rehabilitation outcomes. Given the recent availability of HMD-VR, very few studies have systematically examined how motor learning in an immersive environment using an HMD affects mechanisms of motor adaptation compared to conventional training (CT). Researchers found that discrepancies between real and virtual hand movements during a manipulation task in an HMD-VR versus CT did not affect motor performance^18^, with some suggesting that visuomotor adaptation occurs similarly across virtual and CT environments^19^. However, another study showed that an arm reaching task in both HMD-VR and CT resulted in similar movement profiles but but the HMD-VR system produced longer movement times^20^.

We aimed to examine the mechanisms of visuomotor adaptation in HMD-VR versus CT through use of a visuomotor adaptation paradigm. Visuomotor adaptation is a form of sensorimotor learning that has been widely studied and generally consists of participants learning to adapt, or correct for, an external perturbation^21^,^22^,^23^. For instance, individuals make reaching movements towards a target, and a visual perturbation is applied to the cursor (e.g., the cursor is rotated 45° degree clockwise). Individuals must learn to compensate for this perturbation by a reaching in the opposite direction (e.g., reach 45° degrees counter-clockwise). Learning in this model is largely thought to rely on the formation of an internal model that calculates the difference between anticipated errors of the intended movement and actual errors from sensory feedback, which is then used to plan one’s next movement. This type of learning is often referred to as implicit, or error-based, learning^24^, and is thought to be supported by neural activity in the cerebellum^25^. Recent work, however, has shown that visuomotor adaptation may occur via both an implicit, error-based mechanism and a more cognitive, explicit strategic mechanism^26^. That is, when adapting to a perturbation, an explicit strategy may be used in order to achieve the desired movement outcome. In the example given above, an individual might see that their cursor went 45 degrees clockwise of where they anticipated, and explicitly reason that their next movement should be 45 degrees counter-clockwise to correct for this. Research suggests that both implicit and explicit mechanisms may contribute to visuomotor adaptation^26^.

In this study, we investigated whether visuomotor adaptation in immersive HMD-VR results in similar adaptation effects in CT and whether these effects are achieved through similar mechanisms. We adapted a paradigm used in Taylor *et al*.^26^ to measure both overall adaptation and the balance between implicit and explicit mechanisms in each environment. In this paradigm, participants reported their planned aim and made reaching movements to different targets on a computer screen located in either the virtual or real world (see Fig. 1). After training on the task, a perturbation was introduced; we then measured implicit and explicit learning by comparing the difference between participants’ reported aiming direction and actual hand position. After training with the perturbation, feedback was removed while participants continued to aim at the targets to explore any aftereffects. This paradigm was replicated in both HMD-VR and a conventional training (CT) setting, and participants were randomized to either the HMD-VR group or the CT group. We hypothesized that while participants in either environment should adapt similarly, the mechanisms by which they achieve this adaptation may be different. Based on previous work showing effects of VR on cognitive aspects such as engagement and motivation, we anticipated that the HMD-VR environment may increase participants’ reliance on an explicit, cognitive strategy, while CT would show greater reliance on implicit, error-based mechanisms.

## Results

### Demographics and Subjective Experience Was Not Significant Between Groups

A total of 24 participants (n = 12 per group) participated in this study. There were no significant differences between groups for age, gender, education, or previous VR use between HMD-VR and CT (age: t(16) = 0.16, p = 0.878, HMD-VR: M = 24 ± 5.0; CT: M = 23.75 ± 2.5; gender: χ^2^(1, N = 24) = 0, p = 1; education: χ^2^(3, N = 24) = 7.06, p = 0.07; previous VR use: χ^2^(3, N = 24) = 1.09, p = 0.78). To examine the differences in the physical effects of either environment, participants were asked to fill out post-experiment questionnaires looking at their sense of presence and their sickness level in each environment. While differences in self-reported answers from the presence questionnaire were not significant (realism: t(21.2) = 0.20, p = 0.841, HMD-VR: M = 29.83 ± 8.2, CT: M = 29.08 ± 9.9; possibility to act: t(20.3) = 0.57, p = 0.573, HMD-VR: M = 19.0 ± 4.2, CT: M = 17.83 ± 5.7; possibility to examine: t(19.2) = −1.14, p = 0.269, HMD-VR: M = 11.5 ± 3.1, CT: M = 13.33 ± 4.6; self evaluation of performance: t(21.9) = 0.55, p = 0.586, HMD-VR: M = 9.42 ± 2.2; CT: M = 8.9 ± 2.3), questions addressing the quality of interface approached significance (quality of interface: t(21.2) = 1.95, p = 0.064, HMD-VR: M = 12.92 ± 2.7; CT: M = 10.5 ± 3.3), suggesting that participants in the HMD-VR and CT groups may have felt differences in the HMD-VR versus CT interfaces. Self-reported answers from the simulator sickness questionnaire were also not significantly different between groups (nausea: t(20.9) = −0.67, p = 0.511, HMD-VR: M = 1.67 ± 2.1; CT: M = 2.33 ± 2.7; oculo-motor: t(18.6) = −1.16, p = 0.262, HMD-VR: M = 3.25 ± 2.9; CT: M = 5.08 ± 4.6). These results suggest that individuals in the VR condition did not experience any additional sense of presence or other side effects from being in an immersive virtual environment compared to conventional training during this task, but that there was a trend towards a difference in the quality of the interface.

### HMD-VR and CT Produce Similar Results for Overall Visuomotor Adaptation

The primary outcome measure for the study was target error, which was measured as the difference between the target angle and the participants hand angle. This was used to measure overall adaption throughout each block in the visuomotor adaptation task (Fig. 2A). We compared the target error between training environments while participants reported and reached for targets at baseline (without the perturbation, Block 2: baseline + report) and during the rotation block (with the perturbation, Block 3: rotation + report), as well as after all feedback was removed (Block 4: aftereffect; for a full explanation of the experimental protocol, see Methods). We also compared the reaction time (RT), or the time it took to start a movement from when the target appeared, and the movement time (MT), or the time it took from the start of a movement until the target area was hit. *Baseline* + *Report.* We compared baseline + report (Block 2) performance between groups to ensure that there were no group differences in performance before the visual rotation occurred. There were no significant differences between environments (HMD-VR vs. CT) in target error (t(22) = 0.71, p = 0.48; HMD-VR: M = −1.6° ± 1.5°; CT: M = −1.0° ± 2.3°), RT (t(22) = 0.28, p = 0.78; HMD-VR: M = 1.3 s ± 0.9 s; CT: M = 1.4 s ± 1.1 s), or MT (t(22) = 0.56, p = 0.58; HMD-VR: M = 0.54 s ± 0.50 s; CT: M = 0.67 s ± 0.63 s) during baseline + report. *Rotation* + *Report.* Then, we compared performance between groups during the rotation + report block (Block 3), and again found that there were no significant differences between HMD-VR and CT in target error (t(22) = 1.38, p = 0.18; HMD-VR: M = −0.41° ± 2.0°; CT: M = −2.0° ± 3.4°), RT (t(22) = 0.19, p = 0.85; HMD-VR: M = 0.68 s ± 0.84 s; CT: M = 0.61 s ± 0.76 s), or MT (t(22) = 0.85, p = 0.40; HMD-VR: M = −0.10 s ± 0.24 s; CT: M = −0.24 s ± 0.51 s). In order to understand whether there were differences between groups during early or late adaptation, we also analyzed just the first 6 epochs (early adaptation) and just the last 6 epochs (late adaptation) of the rotation block. For early adaptation, we found that there were no significant differences between HMD-VR and CT in target error (t(22) = 1.33, p = 0.196, HMD-VR: M = −7.20° ± 5.2°, CT: M = −11.84° ± 10.9°), RT (t(22) = −0.32, p = 0.75; HMD-VR: M = 0.65 s ± 0.50 s; CT: M = 0.72 s ± 0.66 s), or MT (t(22) = −0.29, p = 0.77; HMD-VR: M = 1.32 s ± 0.76 s; CT: M = 1.45 s ± 1.27 s). Similarly, during late adaptation, we also found no significant differences between groups in target error (t(22) = 0.90, p = 0.379, HMD-VR: M = 2.92° ± 2.8°, CT: M = 1.99° ± 2.2°), RT (t(22) = 0.38, p = 0.70; HMD-VR: M = 0.69 s ± 1.12 s; CT: M = 0.53 s ± 0.84 s), or MT (t(22) = 0.26, p = 0.80; HMD-VR: M = 1.37 s ± 0.94 s; CT: M = 1.26 s ± 1.11 s). *Aftereffects.* There were also no significant differences between HMD-VR and CT in the no feedback block (Block 4) in target error (t(22) = 0.37, p = 0.71; HMD-VR: M = 6.7° ± 5.7°; CT: M = 7.4° ± 3.9°), RT (t(22) = 0.64, p = 0.53; HMD-VR: M = −0.55 s ± 0.94 s; CT: M = −0.80 s ± 1.00 s), or MT (t(22) = 0.88, p = 0.39; HMD-VR: M = −0.19 s ± 0.26 s; CT: M = −0.37 s ± 0.67 s). Note that RT in the no feedback (Block 4) was much shorter than in either the baseline + report and rotation + report (Blocks 2 and 3, respectively) because participants did not report the aiming direction during the no feedback block. The comparison of target error between environments and across trials is shown in Fig. 3A.

### HMD-VR Relies More on Explicit Learning, CT Relies More on Implicit Learning

We were also interested in the relative contributions of implicit and explicit mechanisms during adaptation. The implicit adaptation (IA) was measured as the difference between the aiming angle and the hand angle, and the explicit component was measured as the self-reported aiming angle. Similar to the analysis of target error, we compared both groups during the baseline, rotation, and no feedback blocks of the visuomotor adaptation task. *Baseline* + *Report.* We compared aiming during the baseline + report (Block 2) and found that there was no significant difference between environments (t(22) = 0.30, p = 0.77; HMD-VR: M = −0.2° ± 0.8°; CT: M = −0.4° ± 1.1°). In addition, we similarly found that there was no significant difference between environments in IA (t(22) = 0.70, p = 0.49; HMD-VR: M = −1.3° ± 1.2°; CT: M = −0.7° ± 2.8°). *Rotation* + *Report.* We compared the effects of training environment on aiming angle and IA during the rotation + report block (Block 3), and found that aiming angle was significantly larger (t(22) = 4.00, p < 0.001) for HMD-VR (M = 41.4° ± 4.1°) compared to CT (M = 33.6° ± 5.4°; Fig. 3B). In addition, the size of the IA was also significantly smaller (t(22) = 3.67, p = 0.001) for HMD-VR (M = 3.3° ± 4.2°) than CT (M = 9.5° ± 4.0°) (Fig. 3C). We also compared the differences between groups during early or late adaptation for aiming angle and IA. For aiming angle, we found there were significant differences between HMD-VR and CT in both early (t(22) = 2.59, p = 0.017, HMD-VR: M = 37.82° ± 6.0°, CT: M = 25.56° ± 15.3°) and late adaptation (t(22) = 3.20, p = 0.004, HMD-VR: M = 41.47° ± 5.1°, CT: M = 34.79° ± 5.2°). We also found significant differences between groups in IA for both early (t(22) = −3.94, p = 0.0007, HMD-VR: M = −0.10° ± 4.3°, CT: M = 7.66° ± 5.3°) and late adaptation (t(22) = −2.44, p = 0.023, HMD-VR: M = 6.59° ± 5.3°, CT: M = 12.29° ± 6.1°). These results suggest that although the overall target error was the same between groups, HMD-VR participants utilized a greater cognitive strategy than CT participants, and CT participants engaged in greater implicit learning than HMD-VR participants (Fig. 3B,C).

## Discussion

In this study, we sought to examine whether visuomotor adaptation, and the mechanisms supporting it, are similar in an immersive virtual reality environment using a head-mounted display compared to a conventional training environment. We show that, while the overall adaptation is similar across both groups, individuals in the virtual reality using a head-mounted display group relied more on an explicit, cognitive strategy to adapt, while individuals in the conventional training group relied more on implicit mechanisms. These findings have implications for how HMD-VR is used to study and manipulate motor learning and rehabilitation.

### Immersive VR Produces Similar Adaptation Effects to Conventional Training

In this study, we show that individuals in both HMD-VR and CT environments are able to successfully adapt to a visuomotor rotation. This is a critical initial validation, as it indicates that either environment can promote, and be used to study, visuomotor adaptation. Visuomotor adaptation has played an importation role in helping scientists uncover the basic neural processes by which the brain supports new motor programs in response to a changing environment^27^,^28^,^29^,^30^. Our findings open up new potential for studying visuomotor adaptation in novel, virtual environments or with virtual manipulations that have not previously been possible in conventional paradigms.

### Adaptation in Immersive VR May Rely on More of a Cognitive Strategy

While the reduction of errors during adaptation occurred similarly across both groups, the mechanisms supporting the adaptation appear to differ. In particular, individuals placed in an immersive VR environment appear to be more reliant on explicit cognitive strategies, while individuals in a conventional, real environment appear to be more reliant on implicit, error-based mechanisms. There may be several potential reasons for this. First, despite the fact that the paradigm and visual environment was designed to be as similar as possible between HMD-VR and CT, the sheer novelty of being in an HMD-VR environment may have increased participants’ attention and engagement and therefore increased reliance on cognitive strategies. To this end, an important follow up study might examine whether individuals with greater experience in HMD-VR continue to utilize more cognitive mechanisms over time, or if familiarization to a HMD-VR environment results in levels of cognitive engagement as CT over time.

It should also be noted that as there were differences between groups in explicit aiming, we would in addition expect to see a difference between groups in the aftereffect magnitude. However, we do not see these difference and only see a decrease in aftereffect magnitude for the CT condition and not for the HMD-VR condition. A recent study by Day *et al*. (2009) found that IA generalizes around the most frequent aiming location and therefore, the aftereffect should be measured at the mean point of the most frequent aim instead of the original target location as is done here. Therefore, one possible explanation for this lack of decreased aftereffect is that HMD-VR may not generalize in the traditional way, but rather in a more global manner. Further studies would be needed to probe whether this interpretation is correct by measuring the aftereffects at different locations.

Overall, it appears that visuomotor adaptation occurs in both HMD-VR and CT, with the strategies employed in each environment to reduce target error differing. It is also worth noting that these results could change if the task was less reliant on cognition. Evidence suggests that endpoint feedback may utilize a more cognitive strategy compared to online feedback, which seems to utilize more of an implicit strategy^26^. This task utilized endpoint feedback, thus biasing individuals towards a more cognitive strategy. Immersive HMD-VR could potentially increase cognitive reliance when cognition contributes to task performance, but these differences may not be as apparent if a cognitive strategy was not as important (e.g., with online feedback). Similarly, if the novelty of the environment affected these results, then individuals with more experience in an immersive VR environment may show patterns in HMD-VR that are more similar to those of CT. These questions, among others, remain to be explored.

### The Future Role of Immersive Virtual Reality in Motor Learning and Rehabilitation

As HMD-VR becomes more ubiquitous, it is likely to become a powerful tool for asking previously inaccessible questions in motor learning and rehabilitation. For instance, learning in dangerous, riskier, or rapidly changing environments can now be explored. However, much research remains to be done to systematically understand the differences between HMD-VR and conventional training on basic aspects of motor learning and adaptation. Our findings provide an initial step towards understanding how immersive VR could be used and interpreted for motor learning studies and clinical rehabilitation. In particular, it is important to note that, while motor learning paradigms may produce similar results in HMD-VR as in conventional training, they may not be accomplished in the same way. HMD-VR may also be a more powerful tool for motor learning and rehabilitation paradigms that engage a strong cognitive component. As researchers and clinicians increase the use of immersive VR for motor rehabilitation, understanding the mechanisms underlying effects produced by this novel environment is critical for designing paradigms that are maximally beneficial to its users.

## Methods

### Participants

A total of 29 individuals were recruited to participate in this experiment. Of this total, 2 individuals were excluded from the study as a result of technical difficulties and 3 individuals were excluded from the study as a result of being beyond two standard deviations away from the mean of target error or aiming direction, resulting in 24 individuals (15 female/9 males, aged: M = 23.9, SD = 3.85; 12 per group) included in the analysis. The 2 participants excluded for technical reasons were in the HMD-VR condition and of the 3 participants who performed poorly, 2 were from the CT condition and 1 was from the HMD-VR condition. Excluding the 3 participants that performed poorly did not significantly change the results of the study. Eligibility criteria included healthy, right-handed individuals with no previous experience with visuomotor adaptation and informed consent was obtained from all subjects. The experimental protocol was approved by the Institutional Review Board at University of Southern California and performed in accordance with the 1964 Declaration of Helsinki.

### Experimental Apparatus

**Figure 1:**
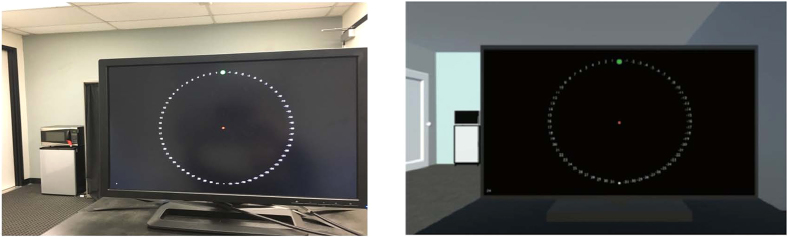
Experimental environments. Top: Visuomotor adaption task in conventional training (CT). Bottom: Visuomotor adaptation task in head-mounted virtual reality (HMD-VR). The environment in virtual reality was designed to emulate the environment of the conventional training condition in the size and lighting of the room, the furniture placed around the room such as a refrigerator and a desk, the computer monitor, and more.

Participants were placed in either a virtual reality environment using the Oculus Rift (DK2) head-mounted display (HMD-VR) or in a conventional training (CT) environment. In both the HMD-VR and CT conditions, we adapted the paradigm used by Taylor and colleagues (2014) in which participants used a digitalized pen and tablet (Wacom Intuos4 Extra Large) to reach for one of eight pseudo-randomized targets located on an upright computer monitor located in either the HMD-VR or CT environment (Fig. 1). Participants were unable to see their hands or forearms in either condition during the duration of the task but visual feedback was provided in the form of a red circular cursor (5 mm diameter) on the computer screen. Observation of one’s hand and forearm movements were occluded in the CT environment with a large box that was place over the tablet, and on top of which the monitor was placed. This box and monitor set-up was replicated in the HMD-VR condition, and there was no virtual body shown in HMD-VR. Movement trajectories were sampled at 60 Hz in both the HMD-VR and CT conditions. In the CT condition, the stimuli were presented on a 24.1 inch, 1920 × 1200 pixel resolution computer monitor (HP) located 23 cm above the tablet. The HMD-VR environment was designed using the game engine development tool, Unity 3D, to replicate the visual characteristics of the CT environment, and was delivered via a head-mounted VR display (Oculus Rift DK2). The HMD-VR environment was designed to emulate features of the physical room where both groups performed the task (e.g., the size and lighting of the room, the furniture, the computer monitor, and more were all similar to the CT environment). The HMD-VR environment was created based on a fixed coordinate system that did not depend on the participant’s head position and all participants were physically seated in the same location for both groups to keep everything consistent.

### Reaching task

**Figure 2:**
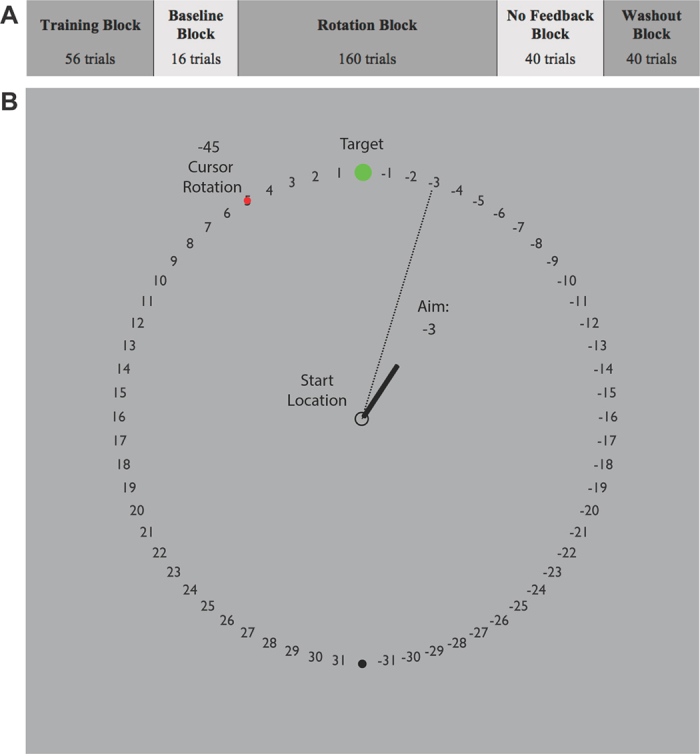
Experimental paradigm. (**A**) Experimental design with blocks and number of trials in each block. (**B**) Visuomotor adaptation task with 45° counterclockwise rotation adapted from Taylor *et al*.^26^. After finding the start circle, participants made quick reaching movements through the targets. Once crossing the outer circle, the endpoint location where their hand crossed the circle appeared as a red circle. During the baseline + report and rotation blocks, participants reported where they were aiming before each reach.

**Figure 3:**
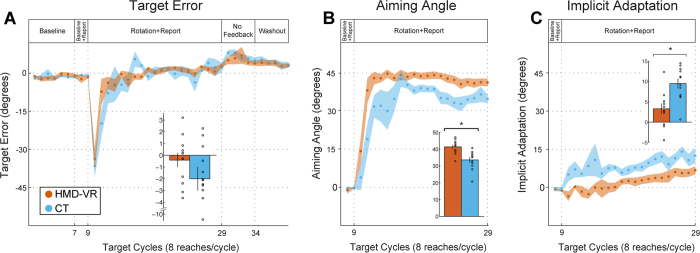
Results between head-mounted virtual reality (HMD-VR) and conventional training (CT). Inset bar graphs show group means and standard errors as well as individual means during the rotation block. (**A**) Target error, measured by subtracting hand angle from target angle, for the HMD-VR group (orange) and the CT group (blue). No significant differences were found between the groups during the baseline + report, rotation + report, or no feedback blocks (Blocks 2–4). (**B**) Aiming angle, measured as the aiming number reported by the participant, for HMD-VR (orange) and CT (blue). The aiming angle was significantly larger (t(22) = 4.00, p < 0.001) for HMD-VR compared to the CT group during the rotation + report block (Block 3). (**C**) Implicit adaptation (aiming angle and rotation subtracted from the target error) for HMD-VR (orange) and CT (blue). The IA was significantly smaller (t(22) = 3.67, p = 0.001) for HMD-VR compared to the CT environment during the rotation + report block (Block 3).

Participants were randomly assigned into one of two conditions resulting in twelve participants per group. Participants completed five distinct blocks that spanned a total of 312 trials (Fig. 2A). At the start of each trial, participants located the starting circle (7 mm diameter) at the center of the screen using a second guiding circle. After staying within the starting circle for 1 second, a cursor appeared (5 mm diameter) along with one of eight pseudo-random green target circles (10 mm diameter). These target circles were located on an invisible ring with a radius of 14 cm and spaced 45° apart (0, 45, 90, 135, 180, −135, −90, −45°). During the initial baseline trials (Block 1: 56 trials), participants were asked to reach towards a target flanked by numbers (Fig. 2B) and complete their reach within 500 milliseconds. Reaction time (RT) was defined as time between when the target appeared and when the cursor exited the starting circle, and movement time (MT) was defined as time between when the cursor exited the starting circle and when the cursor crossed the border of the invisible ring. To encourage faster movements, participants were given a warning via an audio clip saying “Too Slow!” if MT exceeded more than 500 milliseconds. Participants were instructed to make a fast, “slicing” movement through the target. Once they passed the invisible ring, participants received auditory feedback (either a pleasant “ding” if the cursor crossed the target or an unpleasant “buzz” if the cursor did not cross the target). Visual feedback of the cursor’s endpoint position was displayed for 1 second before starting the next block or trial. Excluding no feedback trials (Block 4), participants were given endpoint feedback, in which they were only shown the cursor when their hand crossed the invisible outer ring.

Participants began the paradigm with the baseline block (Block 1: 56 trials) and were instructed to reach for the targets within the set time. In the baseline + report block (Block 2: 16 trials), participants were instructed to continue reaching for the targets within the set time but were additionally asked to explicitly say the number that they were aiming to prior to reaching towards the target. In this way, their explicit aim was recorded, and the difference between their explicit aim and actual hand endpoint position was measured as the implicit component. In the rotation + report block (Block 3: 160 trials), a 45° counterclockwise perturbation was introduced as participants continued the task without any new instructions. In Blocks 4 and 5, the cursor rotation was removed, and numbers no longer flanked the targets. Following the completion of the rotation + report block, participants started the no feedback block (Block 4: 40 trials) where they were asked to reach directly to the targets and told that feedback would not be provided. In the washout block (Block 5: 40 trials), participants continued to aim for the targets without receiving additional instructions. During this phase, both visual and auditory feedback was provided, similarly to the baseline block (Block 1).

### Behavior

Following the completion of the reaching task, participants were asked to complete two questionnaires about their physical reaction to the environment. The first questionnaire, adapted from Witmer & Singer (1998)^31^ and revised by the UQO Cyberpsychology Lab (2004), asked participants a series of questions to gauge their sense of presence in the training environment. Questions were collapsed along five main themes: realism, possibility to act, quality of interface, possibility to examine, and self evaluation of performance. The second questionnaire was adapted from Kennedy, Lane, Berbaum, & Lilienthal (1993)^32^ and revised by the UQO Cyberpsychology Lab (2013) and asked participants a series of questions to gauge their sickness level as a result of being in the training environment. Questions were measured along two main themes: nausea and oculo-motor reactions.

### Movement Analysis

All kinematic data were recorded by Unity 3D (5.1.2, Unity Technologies, San Francisco, CA) for the HMD-VR condition and by MATLAB (MATLAB R2013b, The MathWorks Inc., Natick, MA) for the CT condition. The horizontal direction was set at 0°, and counterclockwise direction was defined as the positive direction. Hand angle was defined as the angle made by the horizontal line (0°) and the line between the origin and the endpoint of the hand. Cursor angle was identical to the hand angle except during the rotation + report block where 45° clockwise was added to the hand angle. For both environments, cursor angle was computed by calculating the intersection point between the invisible ring and a line drawn between the points sampled before and after crossing the invisible ring. Target error was calculated by subtracting hand angle from target angle. Aiming angle was calculated by multiplying the reported aiming number by 5.625 degrees (the number of degrees per number on the invisible ring; 64 numbers in total). Update of the implicit adaptation (IA) was measured by subtracting the participant’s explicitly stated aiming angle from the hand angle. To examine changes in target error, aiming, and IA over the rotation + report block (Block 3), we calculated epochs of 8 movements per epoch and normalized each individual’s performance across the rotation + report trials to the last epoch (8 movements) of baseline + report trials (Block 2). To study whether there were differences in early versus late adaptation, we also analyzed the mean of just the first six epochs (early adaptation) and the mean of just the last six epochs (late adaptation). Finally, we computed the aftereffects of the rotation + report block as the first epoch of no feedback trials (Block 4), normalized to the last epoch of baseline + report trials.

### Statistical Analyses

Statistical analyses for demographics and questionnaires were conducted using R (3.2.2, The R Foundation for Statistical Computing, Vienna, Austria) and statistical analyses for kinematics and motor performance were conducted using MATLAB (MATLAB R2013b, The MathWorks Inc., Natick, MA). To assess differences in the demographics and questionnaires mentioned above either a two sample unpaired t-test for interval data or a chi-squared test for nominal data was performed on each measure across both groups. Similarly, two sample unpaired t-tests were used to examine differences between groups (HMD-VR, CT) on target error, aiming (explicit component), IA (implicit component), and aftereffects.

## Additional Information

**How to cite this article**: Anglin, J. M. *et al*. Visuomotor adaptation in head-mounted virtual reality versus conventional training. *Sci. Rep.* **7**, 45469; doi: 10.1038/srep45469 (2017).

**Publisher's note:** Springer Nature remains neutral with regard to jurisdictional claims in published maps and institutional affiliations.
